# Local Tumor Progression of Hepatocellular Carcinoma After Microwave Percutaneous Ablation: A Preliminary Report

**DOI:** 10.4021/gr401w

**Published:** 2012-01-20

**Authors:** Franco Brunello, Patrizia Carucci, Silvia Gaia, Emanuela Rolle, Paola Rita Brunocilla, Anna Castiglione, Giovannino Ciccone, Mario Rizzetto

**Affiliations:** a Department of Gastro-Hepatology, Azienda Ospedaliera San Giovanni Battista della Citta di Torino, corso Bramante, 88 Turin, Italy; b Departement of Tumor Epidemiology, Azienda Ospedaliera San Giovanni Battista della Citta di Torino, corso Bramante, 88 Turin, Italy

**Keywords:** Tumor, Progression, Hepatocellular carcinoma, Microwave percutaneous ablation

## Abstract

**Background:**

Microwaves (MW) technology is an ablative treatment alternative to radiofrequency (RF) for early stages of hepatocellular carcinoma (HCC) in cirrhotic patients not suitable for surgical resection. It is well known that HCC lesions ≥ 30 mm treated by RF show a high rate of local tumor progression because of residual of unablated neoplastic tissue.

**Methods:**

Aim of this study was to describe a limited experience of MW ablation (9 cirrhotic patients with medium size HCC: 11 lesions, 31 - 50 mm in diameter) treated from June 2009 to May 2010 by one of currently marketed western MW ablation systems and followed up for 2 years. Primary end-point was the probability of local tumor progression at 24 months; secondary end-point was the safety of the procedure.

**Results:**

Radiological response after a single session and re-evaluation of local tumor progression along the time were performed by contrast enhanced computed-tomography at months 1-8-12-24. Early effectiveness rate was 90.1 %. The cumulative incidence of local tumor progression at 1 and 2 years were 36.4% (95% CI 11.2 - 62.7) and 57.6% (95% CI 23.6 - 81.0). We observed a single minor complication of the procedure.

**Conclusions:**

In conclusion, MW ablation system “Amica” has a high rate of primary effectiveness rate but residual of unablated neoplastic tissue induce local tumor progression in about half of the cases during the following 2 years.

## Introduction

Radiofrequency (RF) is nowadays the most popular ablative procedure for the treatment of non-surgical cirrhotic patients with earlier stages of hepatocellular carcinoma (HCC) [1, 2]. Its reliability in obtaining a complete and sustained local response with high effectiveness rates is mainly linked to the lesion diameter although other variables (as site or vicinity to blood vessels) may influence the technical success and primary/secondary effectiveness rates. From a clinical point of view, RF of the smallest (< 20 mm) HCC lesions produces the highest (95 - 98%) effectiveness rates and seems to be associated with an 5-years overall survival similar to that obtained by surgical resection in selected patients [3].

RF ablation of larger lesions, in particular those > 30 mm, exposes to consistent risk of un-complete local response and hence local tumor progression from re-growth of unablated tissue as report many papers on clinical follow-up or histopathologic examination of explanted liver [4-8].

Microwave (MW) technology is an alternative approach of energy deposition inside the neoplastic tissue that induces thermo-lesions by applying electromagnetic waves in the 0.9 - 2.45 GHz range. For many aspects it is quite similar to RF but the temperatures obtained inside the lesion are higher. MW technology has been used since the late nineties in the eastern interventional experience and often called PMCT from “percutaneous microwave coagulation treatment” [9-16]. After the introduction of new devices produced in United States and Europe, MW has gained rising clinical and commercial interest in the western world [17-23].

The related advertising campaigns of marketing claim optimal results of MW for a wide range of diameter (15 - 50 mm) of the lesions. To date, the costs of MW antennas and related generator are higher than those of RF.

We had the opportunity of trying, without extra expense charge for our Institution and hence for a very limited number of cases, four different western MW devices for several cases of HCC nodules and for some neuroendocrine tumors.

Our main interest was directed to the use of MW for the treatment of medium size lesions (31 - 50 mm) HCC. The preliminary experience with a 2.45 GHz MW system produced in Italy showed a high primary effectiveness rate and then a small consecutive series of cases with this dimensional range was treated and successively followed up for 2 years. The results here presented are referred to this system only and do not represent a comparison between different MW ablation systems.

Aims of the study were to give preliminary results about cumulative local progression rate and safety.

## Materials and Methods

During the period June 2009-May 2010 we treated by MW 9 consecutive patients with a total of 11 lesions of HCC. Diagnosis had been performed according to AASLD/EASL guide lines. All the 11 lesions presented 31 - 50 mm diameter (median 38 mm) and were at the first ablative treatment.

All the patients were suffering from liver cirrhosis (Child-Pugh class A/5, A/6 or B/7) and had been considered not suitable for surgical resection. One of them was successively treated by liver transplantation. The median age of the patients was 69.9 years (range: 52.8 - 80.5). Other demographic characteristics are shown in Table 1.

**Table:** Demographic characteristics of the patients

Age (years) – median (range)	69.9 (52.8 – 80.5)
Gender – no (%)	
Male	6 (66.7)
Female	3 (33.3)
Causes of liver cirrhosis – no (%)	
HCV infection	4 (44.4)
HBV infection	1 (11.1)
HBV/HCV co-infection	1 (11.1)
HCV infection / Alcohol	1 (11.1)
HBV infection / Alcohol	1 (11.1)
NASH	1 (11.1)
Child-Pugh score – no (%)	
A/5	5 (55.6)
A/6	3 (33.3)
B/7	1 (11.1)
Previous therapy – no (%)	2 (22.2)

The patients signed a dedicated informed consent approved by the Commission for new medical-surgical devices of our Institution (that includes members of the Ethical Committee): the consent clearly indicated the use of a “non-standard” technical procedure for their ablative procedure.

The MW system used is produced in Italy by Hospital Service Italia, Rome; its trade name is “Amica”. This MW ablation system generates 2.45 GHz MWs with a power of 20 - 80 Watts that are delivered by a 14 gauge antenna. The tip of the antenna-needle is made of ceramic. The system is composed by a MW generator associated with a peristaltic pump that allows the cooling by fresh saline solution in a closed circuit of the antenna-needle. The cooling system reduces the over-heating of the shaft that receives backward reflected energy. Between the tip and the shaft is moreover inserted a particular device, called miniaturized choke, that has been demonstrated to reduce the back diffusion of the MW and to decrease the elliptical shape of the MW-induced thermal lesion [24].

The procedure was performed with the same anesthesiological approach used for RF procedures in our service (deep analgesia-sedation by remifentanil; in some cases propofol was added).

After a local anesthesia of the chest or abdominal wall by infiltration of lidocaine (10 ml) and a small incision of the skin by an 11# scalpel, the needle-antenna was inserted inside the lesion using ultrasound guidance by a skilled operator with long-standing experience of interventional ultrasound.

The power and time of the ablation were defined, according to previous experiences indicated by the official producer. Every case was treated by a single needle-antenna, with 1-3 different consecutive insertions in the same session with power of 60 Watts and for a time of 8-10 minutes each one. All the lesions were treated in a single session.

Evaluation of the response was performed by contrast enhanced computed tomography (CT) scan of upper abdomen at month 1, 8, 12, and 24. A complete response was defined as total disappearance of arterial pathological network associated with an avascular scar of diameter equivalent or larger than the original vital lesion. In the case that was successively given liver transplantation pathological analysis of the lesion treated by MW was done.

Primary end point of this study was cumulative incidence of local tumor progression; secondary end point was safety of the procedure.

Statistical analysis was performed by R software (2.12.1 version). To assess the sustained technical effectiveness of the procedure, the time to local recurrence (represented as cumulative incidence) was evaluated [25], using the 11 treated lesions as units. The cumulative incidence of local recurrence was estimated using the Kaplan-Meier function.

Terminology used in this report is in agreement to that suggested by Goldberg and coworkers in the 2005 report for ablation treatments [26].

## Results

One procedure was complicated by mild pneumothorax associated with a transitory decrease of liver function. The complication recovered without chest drainage. No other complication was observed. Two patients with superficial lesions referred a prolonged pain in the area of insertion for some weeks. The hospitalization length for uncomplicated procedures was 2 days/2 nights. The overall early (4 - 6 weeks after a single session) primary effectiveness rate was 10/11 lesions (90.9%). The lesion with uncomplete response was 40 mm in diameter and the patient harboring this lesion was successively given orthotopic liver transplantation (OLT). The pathological examination of explanted liver described tumor necrosis extending for about 85% of its volume.

At month 8 one local tumor progression was observed in a first patient with a lesion of 50 mm in diameter and others two local tumor progressions were observed at month 12 for two lesions of 35 mm in diameter. During the second year we observed two additional local tumor progressions.

During the 2 years after the initial treatment, new lesions in different segments of the liver were observed in three patients and two patients died after previous local recurrence.

**Figure 1:**
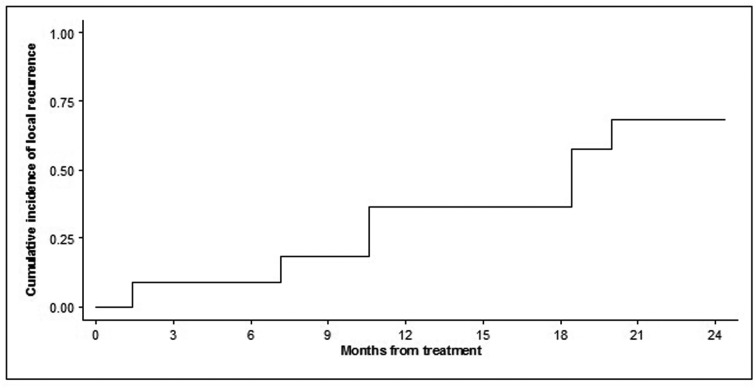
Time to local tumor progression of the 11 treated lesions. Probability is expressed as cumulative incidence of local failure or recurrence from the date of the procedure.

The estimated cumulative incidence of local tumor progression was 36.4% (95% CI 11.2 - 62.7) at month 12 and 57.6% (95% CI 23.6 - 81.0) at month 24 (Fig. 1).

At the 24th month 4 of the 11 lesions maintained a complete local radiological response and 7 out of 9 patients were alive. The patients with tumor progression were treated by chemoembolization or sorafenib.

## Discussion

Accordingly to the AASLD recommendations [1], RF is equivalent to ethanol injection for very small lesions but its technical performance is definitely better for larger lesions. No upper limit for RF is formally defined although the limit of 3 cm seems the highest to obtain good results.

The best technical and clinical results (98.1%) are in fact achieved for lesions with diameter less than 20 mm [3] but its performance as evaluated by radiological means is as high as 95% also for lesions of 21 - 30 mm in diameter [27, 28]. Treatment of non infiltrating larger lesions in the range of 31-50 mm is less satisfactory with an early complete radiological response of 71% [4]. Pathological studies performed on explanted livers harboring HCC lesions ≤ 3 cm previously treated by RF are less optimistic and report complete necrosis in 61.9% and 10% in those larger than 3cm [8]; in other similar reports lesions, > 30 mm show pathological necrosis in no more than 50% [5] or even 29% [29] .

MW has theoretically an important advantage in comparison with RF: the broader primarily active heating not related to electrical tissue conductivity that ensures high temperatures in less time, very limited “heat-sink effect”, no limitation by tissue boiling, dessication, charring. The major disadvantage of MW is represented by the elliptical shape of the thermal lesion due to uncontrolled possible back heating from backward reflexed waves with over-heating of coaxial cable and surrounding non-neoplastic hepatic tissue with potential damage of anatomical structures outside the ablation target.

Our previous experience of unipolar RF by three different type of needles (RITA, Lee Veen, Cool Tip) in 92 HCC lesions treated along 8 years with a range size of 3 - 5 cm in diameter (median 3.7 cm) showed an early radiologic complete response (1 month after 1 - 3 sessions) of 73.9%.

We were then positively surprised by the results of early primary effectiveness (90.9%) that was obtained after a single session whereas RF needs often more sessions. Nevertheless, the follow up revealed that early effectiveness rate was overestimated.

The follow up showed in fact many cases of locally unablated neoplastic tissue that reduced the probability of local tumor progression at about 50% after 2 years.

In general, the MW system used in this study showed no difference in terms of insertion of the needle, guidance, ultrasound visibility and anesthesiologic assistance in comparison to conventional RF. The times of the procedure were reduced (8 - 10 minutes for each exposition *versus* 12 - 24 minutes of RF) in the same range of lesions in our experience with hooked or cooled needles. The post-procedure recovery was not different in comparison to that of the patients treated by RF. A single minor complication was observed.

An obvious limit of our study is the number of the cases treated and our results may consider only a preliminary evidence that MW approach in HCC of 31 - 50 mm could be competitor in comparison with RF.

In conclusion, MW ablation by “Amica” device of HCC nodules of 31 - 50 mm seems to presents a better performance in obtaining the early radiological complete response in comparison to RF but unablated tumor residual are consistent and reveals during the follow up needing additional treatments. Surgical resection, if not contraindicated by liver function or comorbidities, remains today the best curative approach for HCC > 3 cm.
